# Prevalence and Predictors of Gastrointestinal Infections Among Patients With Acute Severe Ulcerative Colitis and Those in Remission: A Cross-Sectional Study

**DOI:** 10.7759/cureus.112376

**Published:** 2026-07-09

**Authors:** Nitesh Bassi, Uday C Ghoshal, Vinod K Dixit, Ujjala Ghoshal, Dawesh Yadav, Tuhina Banerjee, Anurag K Tiwari, Vinod Kumar, Sayan Malakar, Mohita Mahajan, Aakash S Shah, Ishan Mittal, Shishirendu P Parihar

**Affiliations:** 1 Hepatology, Punjab Institute of Liver and Biliary Sciences, Mohali, IND; 2 Gastroenterology, Institute of Medical Sciences, Banaras Hindu University, Varanasi, IND; 3 Gastroenterology, Sanjay Gandhi Postgraduate Institute of Medical Sciences, Lucknow, IND; 4 Microbiology, All India Institute of Medical Sciences, Kalyani, IND; 5 Microbiology, Institute of Medical Sciences, Banaras Hindu University, Varanasi, IND; 6 Dermatology, Government Medical College, Amritsar, IND

**Keywords:** acute severe ulcerative colitis, cytomegalovirus, gastrointestinal infection, inflammatory bowel disease, ulcerative colitis

## Abstract

Background: Gastrointestinal (GI) infections, common in tropical countries, are likely to precipitate acute exacerbations in patients with ulcerative colitis (UC). However, data are scant. Accordingly, we evaluated GI infections in patients with UC and determined their predictors.

Methods: Consecutive patients with acute severe ulcerative colitis (ASUC) and those in remission were evaluated for GI infections by stool microscopy, modified acid-fast stain, culture, and *Clostridioides difficile* toxin A/B/GDH, and cytomegalovirus (CMV) was studied using rectal biopsy by real-time polymerase chain reaction (RT-PCR), histology, and serum immunoglobulin M anti-CMV antibody. Demographic, clinical, and laboratory parameters, along with the frequency and predictors of GI infections, were assessed and compared between the two groups.

Results: Among 158 patients with UC (mean age 35.2 ± 13.0 years; male-to-female ratio 1.3:1; 108 with ASUC and 50 in remission), 79 (50%) had CMV infection (53 detected by RT-PCR alone, one by inclusion bodies, and 25 by both methods). Overall, 92/158 (58.2%) patients had GI infections, of whom 20/158 (12.6%) had multiple infections. CMV infection (70/108 (64.8%) vs. 9/50 (18%); p < 0.001) and overall GI infections (76/108 (70.3%) vs. 16/50 (32%); p < 0.001) were more common in patients with ASUC than in those in remission. *C. difficile* infection was observed in 3.7% of patients with ASUC. CMV coinfection was significantly associated with greater than two courses of systemic corticosteroids, extensive colitis, severe endoscopic inflammation, and deep ulcerations. Patients with concomitant parasitic infections were more likely to have prior azathioprine exposure.

Conclusions: GI infections were more common in patients with ASUC than in those in remission, with CMV being the predominant pathogen; *C. difficile* and parasitic infections were identified in a smaller but clinically relevant proportion of patients. CMV infection was independently associated with prior systemic corticosteroid exposure, severe disease activity, extensive colitis, and deep ulcerations. These findings support routine screening for GI infections, particularly CMV, while considering *C. difficile* and parasitic infections in the diagnostic evaluation of patients with ASUC.

## Introduction

Ulcerative colitis (UC) is a chronic inflammatory disorder of the colon characterized by periods of exacerbation. Approximately 20% of patients experience at least one episode of acute severe ulcerative colitis (ASUC) requiring hospitalization during the course of their disease [1]. Gastrointestinal (GI) infections may precipitate disease flare by disrupting the intestinal microbiome, activating mucosal immune responses, and increasing inflammatory activity in the colon. In the setting of acute exacerbation, enteric infections may act as a complicating factor, may represent the primary precipitating cause, or may simply reflect a commensal state. Various GI infections have been associated with acute exacerbations of UC, including bacterial infections such as *Clostridioides ​​​​​*​*difficile* and *Campylobacter jejuni*, and viral infections such as cytomegalovirus (CMV) [2]. Infectious etiologies should be carefully excluded in patients presenting with a first attack of UC, during exacerbations following remission, and in steroid-nonresponsive disease.

Patients with UC experiencing an acute attack with superadded CMV infection have been shown to have longer hospital stays, higher colectomy rates, and increased mortality [3]. Prompt diagnosis and appropriate treatment of infections can prevent unnecessary exposure to steroids and immunosuppressive therapy. It may also reduce the imprudent use of systemic steroids, which can cause latent enteric infections to manifest and potentially result in fatal outcomes.

Although there remains some debate, many gastroenterologists coprescribe antibiotics during treatment of acute exacerbations of UC. The Indian Society of Gastroenterology, in its consensus guidelines, supports the empirical use of antibiotics in the management of ASUC [4]. The present study aimed to evaluate the frequency of the spectrum of GI infections in patients with ASUC and those in remission and to identify predictors associated with infection.

This article was presented orally at the 65th Annual Conference of the Indian Society of Gastroenterology, ISGCON 2024, December 4-7, 2024, Varanasi.

## Materials and methods

This cross-sectional observational study was performed from 2023 to 2024 on two groups of adult patients (>18 years of age) with UC: the first group of patients with ASUC and the second group of patients with UC in remission, admitted to the Institute of Medical Sciences, Banaras Hindu University, or presenting to outpatient clinics, and were included after satisfying the inclusion and exclusion criteria. Diagnosis of UC was based on clinical, endoscopic, radiological, and histological parameters [5]. Written informed consent was obtained from all study participants. Baseline data for both patient groups were recorded on a standard proforma and captured via a Google Form (Google LLC, Mountain View, CA). All ASUC patients underwent blood and stool sampling and unprepared flexible sigmoidoscopy with biopsy within 24 hours. The following tools were used to assess the severity of UC: Truelove-Witts criteria for ASUC [6] and Mayo score/UC disease activity index for remission [7]. Disease activity was determined by the Ulcerative Colitis Endoscopic Index of Severity (UCEIS) [8]. Disease extent and behavior were classified according to the Montreal classification [9]. For patients presenting with ASUC, extent was determined from the colonoscopy after discharge or the surgical specimen if they underwent colectomy. Rectal biopsies in patients with UC in remission were obtained during clinically indicated colonoscopy (e.g., routine disease assessment/surveillance) and not solely for research purposes. For assessment of infection, three consecutive stool samples of each patient in both groups were subjected to stool microscopy using standard technology, iodine mount, and modified acid-fast stain for protozoa and parasites, including opportunistic pathogens such as coccidian parasites. Stool samples were subjected to conventional bacterial stool culture using standard microbiological techniques routinely employed in our institutional microbiology laboratory for the detection of common enteric bacterial pathogens, along with *C. difficile* Toxin A/B/GDH assay. Rectal biopsies in normal saline were stored at -80°C for assessment of CMV DNA by real-time polymerase chain reaction (RT-PCR). We use the RealStar® CMV PCR Kit 1.0 (altona Diagnostics GmbH, Hamburg, Germany) for the quantitative detection and viral load determination of CMV. The linear range of the RealStar® CMV PCR Kit is 2.420 copies/uL to 2,420,000,000 copies/uL. In addition, histopathology of rectal biopsy was tested for characteristic cytomegalic cells and "owl’s-eye" nuclear inclusion bodies for CMV infection using standard techniques. Immunohistochemistry (IHC) was not performed to diagnose CMV colitis. Serum samples were assessed for immunoglobulin M (IgM) anti-CMV antibodies using standard commercial kits. The objective of our study was to evaluate the prevalence of CMV infection broadly using tissue CMV DNA PCR, histopathological examination for characteristic owl's eye inclusion bodies, and CMV IgM serology.

Inclusion criteria

Consecutive adult patients (≥18 years) with UC presenting with ASUC or those in remission who provided informed consent to participate in the study were included.

Exclusion criteria

Patients aged <18 years, those who had received antibiotics or antiparasitic drugs within the preceding four weeks, and patients with immunodeficiency such as human immunodeficiency virus infection were excluded from the study.

Permissions and approvals

Ethical approval was obtained from the Institutional Ethics Committee of the Institute of Medical Sciences, Banaras Hindu University, Varanasi (2022/EC/3822) and from the All India Institute of Medical Sciences, Kalyani (IEC/AIIMS/Kalyani/certificate/2024/142).

Sample size calculation

The sample size was calculated to detect a difference in the prevalence of GI infections between patients with ASUC and those in remission. Based on earlier Indian studies [10-12], the average frequency of infection was estimated to be 20% in patients with acute UC and 4% in those in remission; the required sample size was calculated using a two-proportion comparison formula with a power of 80% and a significance level of 5%. The estimated minimum sample size was 96 patients in the ASUC group and 48 patients in the remission group. Therefore, a total sample size of at least 144 patients was required. In the present study, 158 patients were included (108 ASUC and 50 remission), which satisfied the calculated sample size requirement.

Statistical analysis

Statistical analysis was performed using the Statistical Package for the Social Sciences software, version 26.0 (IBM Corp., Armonk, NY). Continuous variables were expressed as mean ± standard deviation or median (interquartile range), depending on the distribution. Categorical variables were expressed as frequencies and percentages. Comparisons between the groups were performed using Student’s t-test or Mann-Whitney U test for continuous variables and Chi-square test or Fisher’s exact test for categorical variables. Variables with p < 0.10 in univariate analysis were included in a multivariate logistic regression to identify independent predictors of CMV infection. Adjusted odds ratios with 95% confidence intervals were calculated. Multicollinearity among the independent variables included in the multivariable logistic regression model was assessed using the variance inflation factor (VIF). A VIF value of <5 was considered indicative of the absence of significant multicollinearity. A p value of <0.05 was considered statistically significant.

## Results

Patient population

The mean age of the participants was 35.2 ± 13.0 years, with a male-to-female ratio of 1.3:1. The majority of patients were younger than 30 years, comprising 47.2% of the ASUC group and 50% of the remission group. Patients in the ASUC group had lower body mass index (21.26 vs. 22.12 kg/m²; p = 0.04) and shorter disease duration (23.13 vs. 37.28 months; p = 0.001) compared with those in remission. The ASUC group demonstrated significantly elevated C-reactive protein levels (65.28 mg/dL) compared with the remission group (2.74 mg/dL) (p < 0.001). Additionally, hemoglobin, serum albumin, and total serum protein levels were significantly lower in the ASUC group than in the remission group (p < 0.001). The baseline demographic characteristics and other parameters of patients with ASUC and UC in remission are presented in Table 1.

**Table 1:** Baseline characteristics of patients with ulcerative colitis ASUC: acute severe ulcerative colitis; BMI: body mass index; UCEIS: ulcerative colitis endoscopic index of severity; CRP: C-reactive protein

Variable	ASUC (n = 108)	Remission (n = 50)	Z	p value
Age (years)	35.92 ± 13.95	33.84 ± 10.87	-0.417	0.677
BMI (kg/m²)	21.26 ± 2.27	22.12 ± 1.69	-2.05	0.040
Duration of illness (months)	23.13 ± 24.81	37.28 ± 25.32	-4.085	<0.001
UCEIS	5.97 ± 1.02	0.90 ± 0.46	-10.32	<0.001
Complete Mayo score	11.47 ± 0.85	1.28 ± 0.70	-10.765	<0.001
CRP (mg/dL)	65.28 ± 47.61	2.74 ± 1.69	-9.819	<0.001
Hemoglobin (g/dL)	9.62 ± 2.30	12.38 ± 1.57	-6.624	<0.001
Serum albumin (g/dL)	3.22 ± 0.86	4.16 ± 0.41	-6.807	<0.001
Serum protein (g/dL)	6.09 ± 1.21	7.40 ± 0.69	-6.466	<0.001

The most common disease extent was pancolitis (E3), affecting 63.9% of patients. Left-sided colitis (E2) was present in 32.2%, while proctitis (E1) was observed in a smaller proportion (3.8%). A significantly higher proportion of patients with ASUC had pancolitis (74.1%) compared with those in remission (42%) (p < 0.001).

Extraintestinal manifestations

Extraintestinal manifestations (EIMs) were uncommon in both groups. Deep venous thrombosis was observed only in the ASUC group (5, 4.6%). Axial sacroiliitis was seen in three patients (2.8%) with ASUC and two patients (4.0%) in remission. Peripheral arthritis and episcleritis were noted in two patients (1.9%) each in the ASUC group, but were absent in remission. Pyoderma gangrenosum was observed in one patient (0.9%) with ASUC. Aphthous ulcers were present in six patients (5.6%) with ASUC and two patients (4.0%) in remission, with no significant difference between the groups (p = 0.503).

Frequency of infection

**Figure 1:**
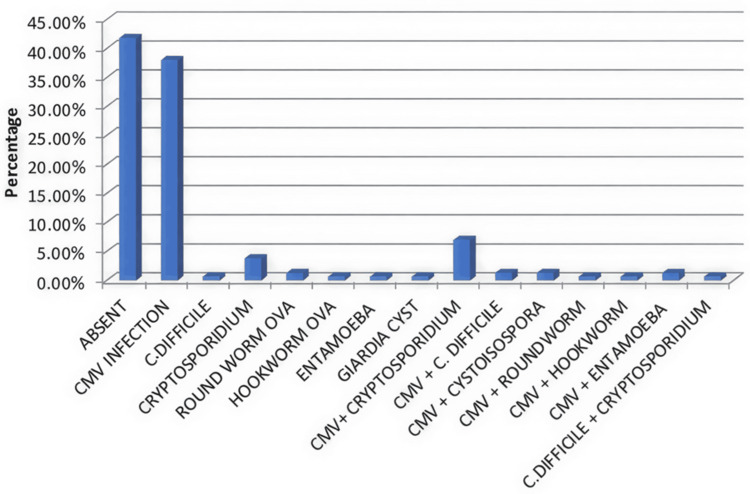
Spectrum of gastrointestinal infections in patients with UC CMV: cytomegalovirus; UC: ulcerative colitis

A substantial proportion (58.2%) of UC patients experienced GI infections. Among these, 45.77% had infection with a single organism, whereas 12.6% had multiple concurrent GI infections. The spectrum of infections included CMV alone (60, 38%), *C. difficile* (1, 0.6%), Cryptosporidium (6, 3.8%), Ascaris (2, 1.3%), hookworm (1, 0.6%), Entamoeba spp. (1, 0.6%), and Giardia (1, 0.6%). Mixed infections included CMV with Cryptosporidium (11, 7%), CMV with *C. difficile* (2, 1.3%), CMV with Cystoisospora (2, 1.3%), CMV with Ascaris (1, 0.6%), CMV with hookworm (1, 0.6%), CMV with Entamoeba spp. (2, 1.3%), and *C. difficile* with Cryptosporidium (1, 0.6%), as shown in Figure 1.

Four (3.7%) patients in the ASUC group had *C. difficile* infection, whereas none of the patients in the remission group had this infection.

CMV infection

Overall, 79 (50%) UC patients had CMV infection, detected by RT-PCR alone in 53 patients, by inclusion bodies alone in one patient, and by both methods in 25 patients. CMV infection (70/108 vs. 9/50) and overall GI infections (76/108 vs. 16/50) were significantly more common among patients with ASUC than among those in remission (p < 0.001). Nearly half (49.36%) of the UC patients exhibited evidence of CMV DNA in rectal biopsy tissue. CMV DNA >250 copies/mg (54.6% vs. 16%) and >1,000 copies/mg (44.4% vs. 10%) were significantly more frequent in ASUC than in remission (p < 0.001).

The mean CMV DNA copy number in rectal biopsy specimens was significantly higher in the ASUC group (7,292,179.48 copies/mg; range 0-75,435,945) compared with the remission group (541.82 copies/mg; range 0-1595.0) (p < 0.001). Among patients with ASUC, 2.8% had positive CMV serology. Additionally, a significant proportion (24.1%) showed evidence of CMV infection on histopathological examination in the form of owl’s eye inclusion bodies, as shown in Table 2.

**Table 2:** Frequency of CMV infection in ulcerative colitis CMV: cytomegalovirus; IgM: immunoglobulin M; ASUC: acute severe ulcerative colitis

CMV detection method	ASUC, n (%)	Remission, n (%)	Chi-square value	p value
CMV DNA ≥250 copies/mg	59 (54.6%)	8 (16%)	28.828	<0.001
CMV DNA ≥1,000 copies/mg	48 (44.4%)	5 (10%)	29.13	<0.001
Histopathology positive	26 (24.1%)	0	14.4	<0.001
CMV IgM positive	3 (2.8%)	0	1.41	0.552

Parasitic infection

**Figure 2:**
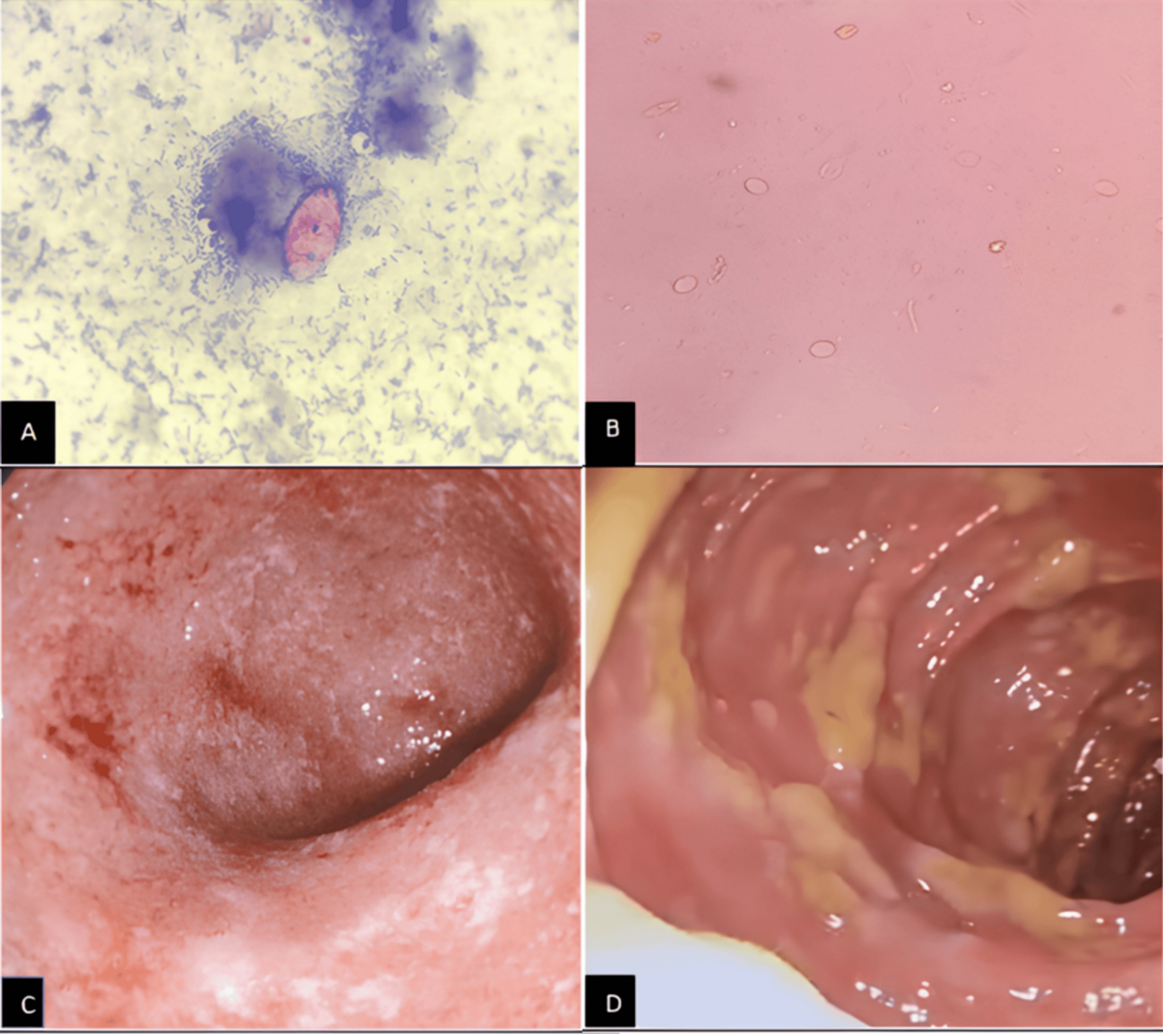
(A) Modified Ziehl-Neelsen stain of stool smear showing acid-fast, elliptical oocyst of Cystoisospora under oil immersion microscopy (100×). (B) Stool microscopy showing cysts of Giardia (40× objective). (C) Colonoscopic view showing multiple superficial ulcers, erosions, and areas of contact bleeding, consistent with active ulcerative colitis. (D) Colonoscopic image showing features of C. difficile infection. The mucosa demonstrates multiple raised yellowish-white plaques (pseudomembranes) adherent to the colonic surface

Nearly one-fifth (18.35%) of patients exhibited evidence of parasitic infection, as detected by standard stool microscopy techniques, including iodine mount and modified acid-fast staining. Cryptosporidium was the most common infection, affecting 11.39% (18) of patients. Other parasites, including Cystoisospora (1.27%, 2), roundworm (1.9%, 3), hookworm (1.27%, 2), Entamoeba spp. (1.9%, 3), and Giardia (0.63%, 1), were detected in smaller proportions, as shown in Figures 1, 2.

The prevalence of parasitic infections was comparable between patients with ASUC (17.6%) and those in remission (20%), with no statistically significant difference observed. Similarly, there were no statistically significant differences in the distribution of these infections between the two groups (p = 0.27). In all 158 UC patients, no enteropathogenic organism was identified on stool culture.

Predictors of infection

**Figure 3:**
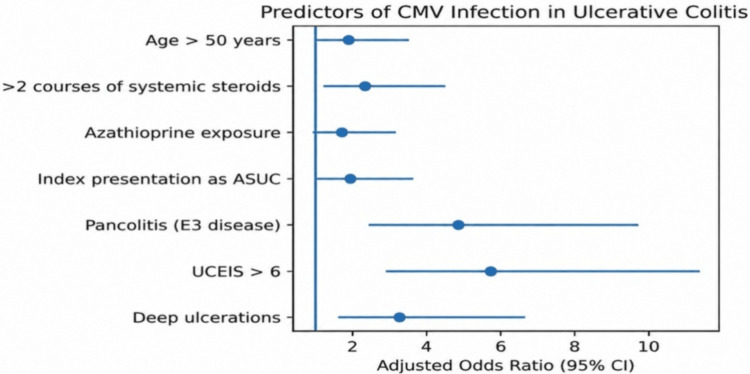
Forest plot of adjusted odds ratio ASUC: acute severe ulcerative colitis; CMV: cytomegalovirus; UCEIS: ulcerative colitis endoscopic index of severity; CI: confidence interval

Patients with CMV coinfection demonstrated a higher likelihood of azathioprine therapy (27.8% of patients; p = 0.08), more than two courses of systemic steroids (51.9%; p = 0.05), initial presentation as ASUC (22.8%; p = 0.09), and older age (>50 years) (17.7%; p = 0.05). Moreover, patients with CMV coinfection were significantly more likely to have extensive colitis (E3 disease in 81%; p < 0.001), higher clinical disease activity (complete Mayo score of 12 in 70.9%; p < 0.001), severe endoscopic inflammation (UCEIS > 6 in 68.4%; p < 0.001), and a higher prevalence of deep ulcerations on colonoscopy (51.6%; p < 0.001), as shown in Tables 3, 4 and Figure 3.

**Table 3:** Predictors of CMV infection (univariate analysis) CMV: cytomegalovirus; UCEIS: ulcerative colitis endoscopic index of severity

Variable	CMV absent	CMV present	Chi-square value	p value
Age >50 years	6 (7.6%)	14 (7.7%)	3.641	0.05
Azathioprine exposure	13 (6.5%)	22 (27.8%)	2.973	0.08
Two steroid courses	29 (36.7%)	41 (51.9%)	4.912	0.05
Pancolitis	37 (46.8%)	64 (81.0%)	20.288	<0.001
UCEIS >6	18 (22.8%)	54 (68.4%)	47.597	<0.001
Complete Mayo score	20 (25.3%)	56 (68.4%)	41.218	<0.001
Deep ulcerations	8 (10.1%)	25 (31.6%)	11.070	<0.001

**Table 4:** Multivariate logistic regression for predictors of CMV infection in ulcerative colitis OR: odds ratio; CI: confidence interval; CMV: cytomegalovirus; UCEIS: ulcerative colitis endoscopic index of severity; ASUC: acute severe ulcerative colitis

Variable	Adjusted OR	95% CI	p value
Age >50 years	1.89	1.01-3.52	0.045
Azathioprine exposure	1.71	0.92-3.17	0.088
>2 courses of systemic steroids	2.34	1.21-4.51	0.012
Pancolitis (E3 disease)	4.86	2.43-9.72	<0.001
UCEIS >6	5.74	2.90-11.38	<0.001
Index presentation as ASUC	1.94	1.03-3.64	0.039
Deep ulcerations	3.27	1.61-6.66	0.001
Complete Mayo score = 12	4.95	2.44-10.03	<0.001

Several variables included in the multivariable model, such as the Complete Mayo score, UCEIS, pancolitis, deep ulcerations, and ASUC status, reflect related aspects of disease severity and may therefore exhibit conceptual overlap. However, assessment of multicollinearity demonstrated acceptable VIF values, suggesting that this overlap did not materially affect the stability of the regression model. Nevertheless, the potential influence of residual collinearity should be considered when interpreting the adjusted estimates.

Among patients with UC, 20 (12.6%) had concurrent infection with coccidian parasites (Cryptosporidium and Cystoisospora), while nine (5.69%) had other parasitic infections (including roundworm, hookworm, Entamoeba spp., and Giardia) identified on stool examination. No statistically significant differences were observed among the three patient groups (those without parasitic infection, those with coccidian parasites, and those with other parasitic infections) with respect to age, sex, exposure to immunosuppressive therapy or systemic steroids, timing of steroid initiation, index presentation as ASUC, clinical and endoscopic disease severity, or the presence of deep ulcerations. However, patients with UC and concomitant parasitic infections were more likely to have received azathioprine therapy compared with those without parasitic infections (p = 0.048).

## Discussion

In this study, we evaluated the frequency and spectrum of GI infections among patients with UC and identified predictors of infection. Our findings demonstrate that GI infections are highly prevalent among patients with UC, particularly among those presenting with acute severe disease. Prompt identification of infections before escalation of therapy may help clinicians optimize treatment decisions.

In a study from a tertiary care center in Chandigarh, infection with protozoal or bacterial pathogens and/or the presence of *C. difficile* toxin was detected in eight (32%) patients with acute exacerbation of UC and one (4%) patient with disease in remission (p < 0.05) [10]. In our study, a substantial proportion (58.2%) of patients had GI infections, and 12.6% had multiple concurrent infections. The prevalence of GI infections was significantly higher in the ASUC group (70.4%) compared with the remission group (32.0%) (p < 0.001). Similarly, a study from Vellore reported that coinfection with enteric pathogens, including parasites, CMV, and *C.* *difficile*, was significantly associated with moderate-to-severe UC [13]. Nearly half of the patients with UC in our study had evidence of CMV infection, and CMV infection was significantly more common in patients with ASUC. Similar findings were reported in a study from Delhi, which detected CMV DNA in mucosal biopsies of 59% of patients with active UC [14]. In our cohort, 79 patients had CMV infection: detected by RT-PCR alone in 53 patients, by inclusion bodies alone in one patient, and by both methods in 25 patients. Histopathological evidence of CMV infection (owl’s eye inclusion bodies) was observed in 24.1% of patients with ASUC, whereas none of the patients in remission showed CMV infection on histopathology (p < 0.001). A study from Lucknow reported CMV infection in 15.8% of patients with inflammatory bowel disease (IBD), with PCR, IgM serology, and histopathology sensitivities of 80%, 60%, and 10%, respectively [12]. In our study, 2.8% of patients with ASUC had positive CMV IgM serology, suggesting systemic infection. Although CMV IgM may indicate acute systemic infection or viral reactivation, its limited sensitivity precludes meaningful conclusions regarding its clinical utility in CMV colitis [15]. CMV DNA >250 copies/mg (54.6% vs. 16%) and >1,000 copies/mg (44.4% vs. 10%) were significantly more frequent in ASUC than in remission (p < 0.001). A previous study involving 42 patients with acute UC demonstrated that CMV DNA levels exceeding 250 copies/mg were predictive of steroid failure and nonresponse to infliximab and cyclosporine [16]. In contrast, 16% of patients in remission in our study also had CMV DNA levels exceeding 250 copies/mg. Another study from Delhi reported that a high CMV DNA load (>2,000 copies/mg) predicted steroid failure in ASUC with a sensitivity of 53% and specificity of 90% [14]. However, a universally accepted threshold for CMV DNA levels in rectal biopsy specimens for the diagnosis of CMV disease has not yet been established. This is largely due to challenges in standardizing quantitative assays and expressing viral load in uniform units across different laboratories [17-19].

In the present study, 3.7% of patients with ASUC had *C. ​​​​​​difficile* infection. Comparable findings have been reported in an Indian cohort, in whom 3.4% of patients with UC tested positive for *C. difficile* [13]. In contrast, another study from Delhi reported a much lower prevalence, with only 0.65% of patients with ASUC testing positive for *C. difficile* toxin, despite the presence of several predisposing risk factors [20]. In our cohort, 65.8% of patients received systemic corticosteroids, 44.3% had undergone more than two courses of steroids, and 22.1% received azathioprine therapy. However, none of the patients had a history of recent antibiotic or antiparasitic drug use or hospitalization within the preceding four weeks.

Nearly one-fifth of patients (18.35%) demonstrated evidence of parasitic infection. The relatively high prevalence observed in this study may be attributed to contaminated drinking water and suboptimal personal hygiene, as most of the identified parasites are waterborne pathogens. Previous studies have reported a high prevalence of parasitic infections in active UC [11], and they were significantly associated with moderate-to-severe disease activity [13].

CMV coinfection in our study was associated with higher rates of prior azathioprine use, more than two courses of systemic steroids, initial presentation as ASUC, and older age. Additionally, CMV coinfection showed significant associations with extensive colitis, higher clinical disease activity, severe endoscopic inflammation, and the presence of deep ulcerations. Similar observations have been reported previously, where patients with IBD and CMV infection were more likely to be female, have pancolitis, exhibit histological disease activity, and receive azathioprine therapy [12]. Furthermore, several studies have demonstrated that the use of immunomodulators is significantly associated with the development of CMV disease in IBD [21-23]. In the present cohort, patients with UC and CMV coinfection had a higher prevalence of deep ulcerations on colonoscopy (51.6%; p = 0.001). Similar findings have been described in other studies, where deep, well-defined punched-out ulcerations were observed in approximately 70%-80% of patients with CMV colitis [24,25].

No clinical or demographic factors were significantly associated with predisposition to parasitic infections in this study, except that patients with UC and concomitant parasitic infections were more likely to have received azathioprine therapy (p = 0.048). Similar findings have been reported previously, where no significant association was observed between protozoal infection and clinical variables such as gender, disease extent, EIMs, medical therapy, or disease activity [26]. The absence of enteropathogenic organisms on stool culture may reflect the inherent limitations of conventional culture techniques, which have suboptimal sensitivity and detect only a limited spectrum of bacterial pathogens. Additionally, preanalytical factors related to specimen collection, transport, or processing may have contributed to the negative results. These findings underscore the potential value of more sensitive molecular assays, such as multiplex stool PCR, for detecting enteric pathogens in patients with ASUC. Although the study provides important insights, it has several limitations. This study has several limitations. First, it was conducted at a single center with a relatively small sample size, limiting the generalizability of the findings; therefore, larger prospective multicenter studies are warranted. Second, the observational design precludes establishing a causal relationship between GI infections and UC disease flares. Third, IHC, the gold standard for diagnosing CMV colitis, and stool multiplex PCR for sensitive detection of enteric pathogens were not performed because these facilities were unavailable at our institution. Additionally, there is no universally accepted cutoff for tissue CMV DNA. Although thresholds of >250 and >1,000 copies/mg were selected based on previous studies, their clinical significance requires further validation. Finally, clinical outcomes according to infection status were not evaluated; therefore, further prospective studies are needed to determine the impact of these infections on treatment response and long-term clinical outcomes.

## Conclusions

GI infections are common among patients with UC, particularly those presenting with acute severe disease. CMV infection represents the most frequent pathogen and is strongly associated with greater than two times exposure to systemic steroids, severe disease activity, extensive colitis, and deep ulcerations. Our study showed a high prevalence of parasitic infection in patients of UC, which may be due to the consumption of contaminated drinking water and poor personal hygiene practices, as the majority of parasitic infections in this study are water‑borne infections. Patients with UC and concomitant parasitic infections were more likely to have received azathioprine treatment. Routine screening for GI infections, including CMV, should be considered in patients with ASUC to optimize management and improve clinical outcomes.
